# 3D-QSAR Studies on a Series of Dihydroorotate Dehydrogenase Inhibitors: Analogues of the Active Metabolite of Leflunomide

**DOI:** 10.3390/ijms12052982

**Published:** 2011-05-10

**Authors:** Shun-Lai Li, Mao-Yu He, Hong-Guang Du

**Affiliations:** College of Science, Beijing University of Chemical Technology, 15 Beisanhuan East Road, Chaoyang District, Beijing 100029, China; E-Mail: wulianhmy2008@163.com (M.-Y.H.)

**Keywords:** DMARDs design, DHODH inhibitors, 3D-QSAR, SOMFA

## Abstract

The active metabolite of the novel immunosuppressive agent leflunomide has been shown to inhibit the enzyme dihydroorotate dehydrogenase (DHODH). This enzyme catalyzes the fourth step in *de novo* pyrimidine biosynthesis. Self-organizing molecular field analysis (SOMFA), a simple three-dimensional quantitative structure-activity relationship (3D-QSAR) method is used to study the correlation between the molecular properties and the biological activities of a series of analogues of the active metabolite. The statistical results, cross-validated r_CV_^2^ (0.664) and non cross-validated r^2^ (0.687), show a good predictive ability. The final SOMFA model provides a better understanding of DHODH inhibitor-enzyme interactions, and may be useful for further modification and improvement of inhibitors of this important enzyme.

## 1. Introduction

The dihydroorotate dehydrogenase (DHODH) ia an essential mitochondrial enzyme that catalyzes the flavin mononucleotide-dependent formation of orotic acid, a key step in *de novo* pyrimidine biosynthesis [1,2]. This enzyme is an attractive chemotherapeutic target in various pathogens, such as *Plasmodium falciparum* and *Helicobacter pylori*, and for the treatment of human disease, such as cancer, malaria and rheumatoid arthritis [3–5].

All potent inhibitors of DHODH published to date bind to the putative ubiquinone binding channel and display beneficial immunosuppressive and antiproliferative activities, shown to be most pronounced during T-cell proliferation [6]. Brequinar and leflunomide are two examples of low-molecular weight inhibitors of DHODH that have been in clinical development [7–9]. Leflunomide is now marketed as a treatment for rheumatoid arthritis. A series of analogues of the active metabolite of an immunosuppressive agent leflunomide have also been synthesized and found to inhibit DHODH [10].

Quantitative structure-activity relationships are the most important applications of chemometrics, giving useful information for the design of new compounds acting on a specific target. Quantitative structure-activity relationship (QSAR) attempts to find a consistent relationship between biological activity and molecular properties. Thus, QSAR models can be used to predict the activity of new compounds. Although there has been much interest in synthesis of various inhibitors of DHODH, there have been few QSAR studies of DHODH inhibitors [10–13]. Kuo [10] and Ren [11] have even reported the structure-activity relationships (SAR) and quantitative structure-activity relationship (2D-QSAR) of this series of analogues, respectively.

The self-organizing molecular field analysis (SOMFA) [14] is a simple 3D-QSAR technique, which has been developed by Robinson *et al*. The method has similarities to both comparative molecular field analysis (CoMFA) [15] and molecular similarity studies. Like CoMFA, a grid-based approach is used; however, SOMFA only uses steric and electrostatic maps, which are related to interaction energy maps, no probe interaction energies need to be evaluated. The weighting procedure of the grid points by Mean-Centered-activity is an important ingredient of the SOMFA procedure. Like the similarity methods, it is the intrinsic molecular properties, such as the molecular shape and electrostatic potential, which are used to develop the QSAR models.

A SOMFA model could suggest a method of tackling the all-important alignment, which all 3D-QSAR methods have faced. The inherent simplicity of this method allows the possibility of aligning the training compounds as an integral part of the model derivation process and of aligning prediction compounds to optimize their predicted activities.

In a recent study, leflunomide has been found to exhibit some dose-dependent side effects in a small number of patients [16]. The purpose of this paper is to describe the application of self-organizing molecular field analysis, SOMFA, on the analogues of the active metabolite of leflunomide, to analyze the three-dimensional quantitative structure-activity relationships (3D-QSAR) and to determine the structural requirements of this series of analogues for optimum activity. The 3D-QSAR together with the modeling studies will provide a more precise elucidation of the molecular forces involved in the DHODH inhibitor-enzyme interactions, and may be useful for further modification and improvement of inhibitors of this important enzyme.

## 2. Materials and Methods

### 2.1. Data Sets

The biological activities of analogues of the active metabolite of leflunomide were taken from the papers by Kuo *et al*. [10]. Not every compound from Kuo’s paper was included in the 3D-QSAR analysis because of the lack of parameters (6 compounds) and the exact IC_50_ values (IC_50_ > 10^5^ nM for 6 compounds). All analogues were classified into two subgroups according to the substituents at their two different positions, 42 aromatic substituted analogues and 12 side chain 3-substituted analogues.

Fifty-three analogues are divided into two sets. The training set of 42 molecules with structures are shown in Table 1 and their enzyme inhibitory activities expressed as log(1/IC_50_) are shown in Section 3. The predictive power of the models is evaluated using a test set of 11 molecules whose structures are also shown in Table 1 and activities will be shown in Section 3.

Two sets of molecules are selected in order to elucidate convenient models for the predictive discrimination between these various activities.

### 2.2. Molecular Modeling and Alignment

The three-dimensional structures of the analogues were constructed with the ArgusLab 4.01 [17] according to the conformations of active metabolite A771726 (Compound 43) from PDB 1d3h [18], running on an AMD Athlon 64 X2 Dual Core Processor 3600 + CPU/Microsoft Win XP platform.

Unless otherwise indicated, parameters are default. Full geometry optimizations are performed first by molecular mechanics MM2, and then optimized by PM3 semi-empirical method in the ArgusLab software. The final active conformations are then performed RMS overlapping and fitted with the compound 43 as a reference. Two different alignments are selected to define overlap. The atom numbers and corresponding sequence for each alignment are defined in Table 2.

**Figure 1.**
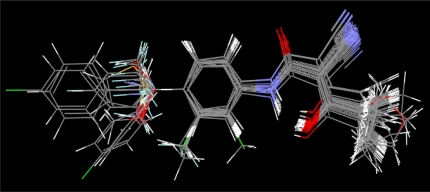
Superposition of active analogues structures according to alignment 1.

**Figure 2.**
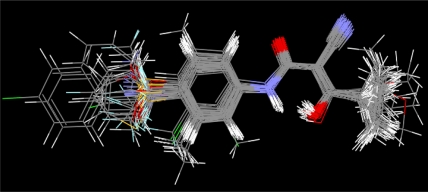
Superposition of active analogues structures according to alignment 2.

According to two alignment of the final active conformations of analogues, these compounds are then performed SOMFA analysis. The superposition of active analogues structures according to alignment 1 (considering phenyl ring similarity) has been shown in Figure 1, the superposition of active analogues according to the alignment 2 (considering all skeleton similarity) has also been shown in Figure 2. Using VEGA software [19], the final overlayed geometries are converted into CSSR file format, the only file format which the SOMFA2 program can accept to process a SOMFA analysis.

### 2.3. SOMFA 3D-QSAR Models

In the SOMFA study a 40 × 40 × 40 Å grid originating at (−20, −20, −20) with a resolution of 0.5 Å, is generated around the aligned compounds, and all compounds have been assigned charges by the MNDO hamiltonian semi-empirical method according to our previous works [20,21]. 12 different models using different enzyme, compound subgroups and alignment under exploration are presented in Table 3.

For all of the studied compounds, shape and electrostatic potential are generated. To sum up the predictive power of these two properties into one final model, we combine their individual predictions using a weighted average of the shape and electrostatic potential based QSAR, using a mixing coefficient (c_1_) as illustrated in Equation 1 [14].

(1) $$\text{Activity}\;=\;{\text{c}}_{1}{\text{Activity}}_{\text{shape}}\;+\;(1\;-\;{\text{c}}_{1}){\text{Activity}}_{\text{ESP}}$$

Clearly, multiproperty predictions could have been obtained through multiple linear regression.

Using Equation 1 instead gives greater insight into the resultant model by allowing the study of the variation in predictive power with different values of c_1_.

With the highest value of r^2^, the SOMFA models then are derived by the partial least squares (PLS), implemented in SPSS software [22] with cross-validation.

The predictive ability of the model is quantitated in terms of r_CV_^2^ which is defined in Equation 2.

(2) $${\text{r}}_{\text{CV}}^{2}\;=\;(\text{SD}\;-\;\text{PRESS})/\text{SD}$$

where PRESS = σ (Y_pred_ − Y_actual_) and SD = σ (Y_actual_ − Y_mean_).

SD is the sum of squares of the difference between the observed values and their meaning and PRESS is the prediction error sum of squares. The final models are constructed by a conventional regression analysis with the optimum value of mixing coefficient (c_1_) equal to that yielding the highest r^2^ and r_CV_^2^ value according to Equation 2.

## 3. Results and Discussion

SOMFA, a novel 3D-QSAR methodology, is employed for the analysis with the training set composed of 42 various compounds, from which biological activities are known. Statistical results of 12 SOMFA models are summarized in Table 3.

A cross-validated value r_CV_^2^ which is obtained as a result of PLS analysis serves as a quantitative measure of the predictability of the SOMFA model. We find that the quality of the QSAR model was dependent upon the alignment and number of molecules. The model overlayed using alignment 2 shows higher r_CV_^2^ values than using the model of alignment 1, and the model of subgroups shows higher r_CV_^2^ values than the model of all analogues.

Among the models tested from all analogues, good cross-validated correlation coefficient r_CV_^2^ values (0.664), moderate non-cross-validated correlation coefficient r^2^ values (0.687) proves a good conventional statistical correlation which have been obtained, and we also find that the resultant SOMFA model have a satisfying predictive ability.

**Figure 3.**
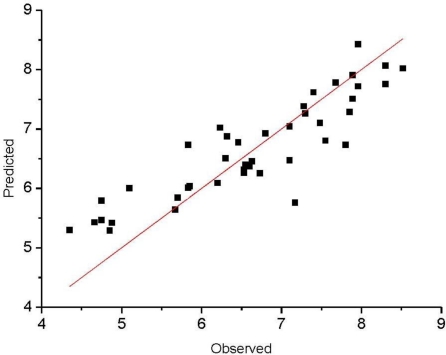
Observed *versus* predicted activities (Rat DHODH) in the training set.

The observed and predicted activities of the training set are reported in Table 4. Figure 3 shows a satisfying linear correlation and moderate difference between observed and predicted values of molecules in the training set.

**Figure 4.**
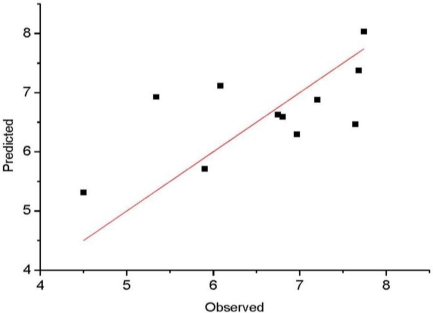
Observed *versus* predicted activities (Rat DHODH) in the test set.

It is well known that the best way to validate a 3D-QSAR model is to predict biological activities for the compounds forming the test set. The SOMFA analysis of the test set composed of 11 compounds is reported in Table 5. Most of the compounds in the test set show satisfying correlation between observed and predicted values in Figure 4. We find that two compounds of test set (compound 10 and 15) always have large residuals, and could be classified as outliers. This is true for both rat and mouse DHODH models, there may be more complicated structure-activity relationships in these two compounds. The statistical parameters r_pred_^2^ of test compounds excluding compound 10 and 15 are also summarized in Table 3; all the models performed well (r_pred_^2^ > 0.5) in the activity prediction of most test compounds.

**Figure 5.**
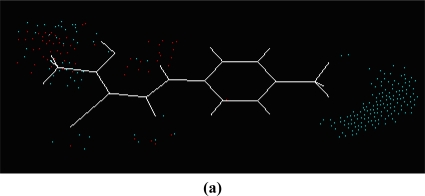

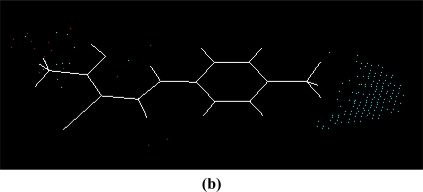
The electrostatic potential master grid with compound 43, red represents areas where postive potential is favorable, or negative charge is unfavorable, blue represents areas where negative potential is favorable, or postive charge is unfavorable. **(a)** Rat DHODH and **(b)** Mouse DHODH.

**Figure 6.**
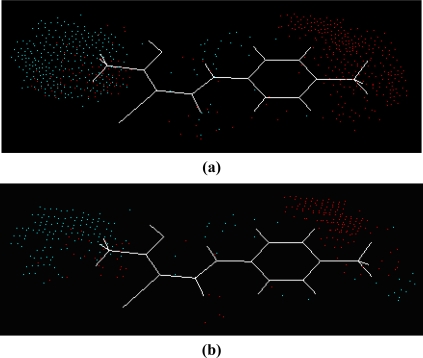
The shape master grid with compound 43, red represents areas of favorable steric interaction; blue represents areas of unfavorable steric interaction. **(a)** Rat DHODH and **(b)** Mouse DHODH.

SOMFA calculation for both shape and electrostatic potentials are performed, then combined to get an optimal coefficient c_1_ = 0.766 according to Equation 1. The master grid maps derived from the best model is used to display the contribution of electrostatic potential and shape molecular field. The master grid maps give a direct visual indication of which parts of the compounds differentiate the activities of compounds in the training set under study. The master grid also offers an interpretation as to how to design and synthesize some novel compounds with much higher activities. The visualization of the potential master grid and shape master grid of the best SOMFA model is showed in Figure 5 and Figure 6 respectively, with compound 43 as the reference.

Each master grid map is colored in two different colors for favorable and unfavorable effects. In other words, the electrostatic features are red (more positive charge increases activity, or more negative charge decreases activity) and blue (more negative charge increases activity, or more positive charge decreases activity), and the shape feature are red (more steric bulk increases activity) and blue (more steric bulk decreases activity), respectively.

It can be seen from Figure 5 and Figure 6 that the electrostatic potential and shape master grid for Rat DHODH are very similar to that for Mouse DHODH. Because Rat DHODH have structural similarities to Mouse DHODH, so active analogues have the same or a similar 3D-QSAR to them.

SOMFA analysis result indicates the electrostatic contribution is of a low importance (c_1_ = 0.766). In the map of electrostatic potential master grid, we find a high density of blue points around the substituent R_1_ at the phenyl ring, which means some electronegative groups are favorable. Meanwhile, the SOMFA shape potential for the analysis is presented as master grid in Figure 6. In this map of important features, we can find a high density of red points around the substituent R_1_ and R_2_ at the phenyl ring, which means a favorable steric interaction; simultaneously, we also find a high density of blue points outside substituent R at the 3-substituted side chain, where an unfavorable steric interaction may be expected to enhance activities. Generally, the medium-sized electronegative potential substituent R_1_ and R_2_ (benzene ring with electron-withdrawing groups, pyridine ring, for example) at the phenyl ring increases the activity, the small-sized substituent R (methyl, ethyl, for example) at the 3-substituted side chain increases the activity.

All analyses of SOMFA models may provide some useful information in the design of new active metabolite analogues of leflunomide.

## 4. Conclusions

We have developed predictive SOMFA 3D-QSAR models for analogues of the active metabolite of Leflunomide as anti-inflammatory drugs. The master grid obtained for the various SOMFA models’ electrostatic and shape potential contributions can be mapped back onto structural features relating to the trends in activities of the molecules. On the basis of the spatial arrangement of the various electrostatic and shape potential contributions, novel molecules are being designed with improved activity.

## Figures and Tables

**Table 1.** Chemical structures of active metabolite analogues of leflunomide.

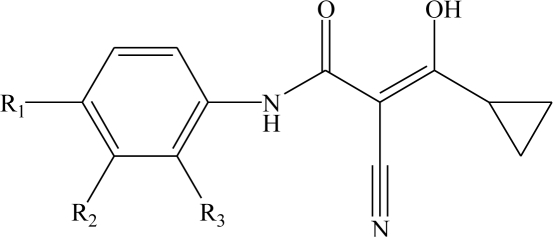							
**Compd No.**	**R_1_**	**R_2_**	**R_3_**	**Compd No.**	**R_1_**	**R_2_**	**R_3_**
1	H	H	H	29	Cl	CH_3_	H
2	CH_3_	H	H	30*	Cl	H	CH_3_
3	CF_3_	H	H	31	CH_3_	Cl	H
4	H	CF_3_	H	32	Br	CH_3_	H
5*	Cl	H	H	33	CN	CH_3_	H
6	H	Cl	H	34	CF_3_S	CH_3_	H
7	H	H	Cl	35*	CF_3_O	CH_3_	H
8	Br	H	H				
9	CN	H	H	36	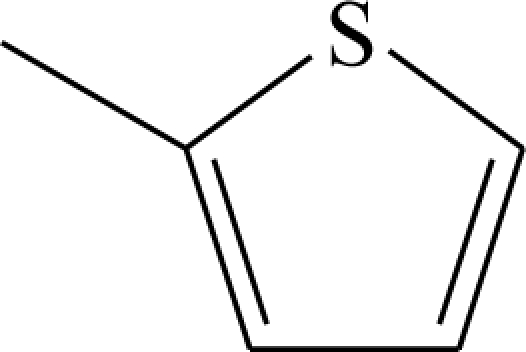	H	H
10*	-CH_2_CN	H	H				
11	CF_3_S	H	H				
12	CF_3_SO	H	H	37	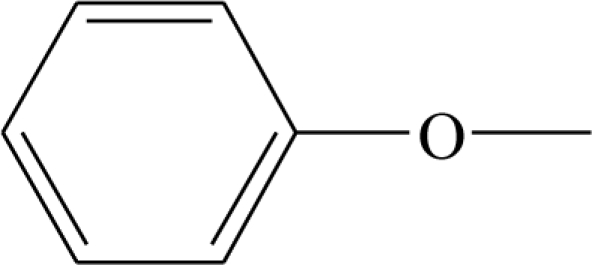	H	H
13	CF_3_SO_2_	H	H				
14	CH_3_S	H	H				
15*	CH_3_SO	H	H	38	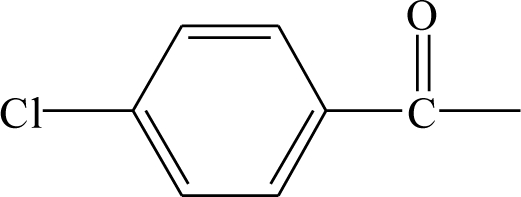	H	H
16	CH_3_SO_2_	H	H				
17	CF_3_O	H	H				
18	CH_3_O	H	H	39	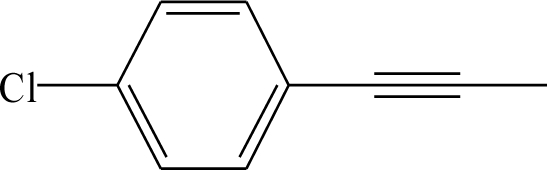	H	H
19	OH	H	H				
20*	NO_2_	H	H				
21	H_2_N	H	H	40*	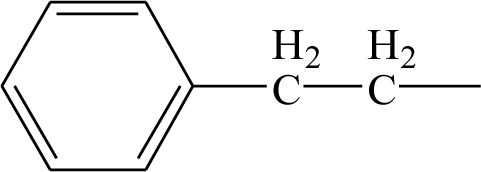	H	H
22	CH_3_CO	H	H				
23	H_2_NCO	H	H				
24	HOOC-	H	H	41	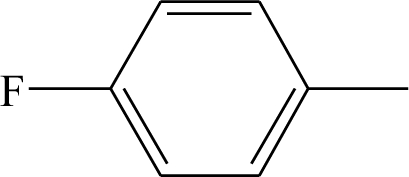	H	H
25*	CH_3_O_2_C-	H	H				
26	CF_3_	CH_3_	H				
27	CF_3_	C_2_H_5_	H	42	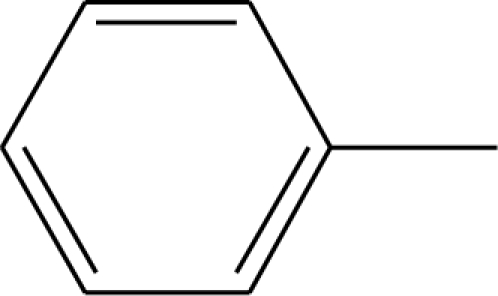	H	H
28	C_2_F_5_	CH_3_	H				

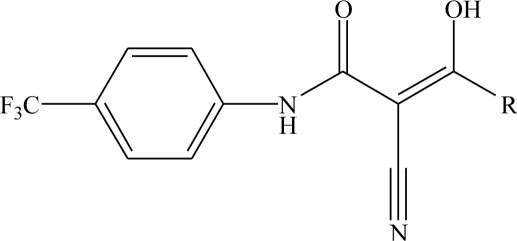			
**Compd No.**	**R**	**Compd No.**	**R**
43	-CH_3_	48	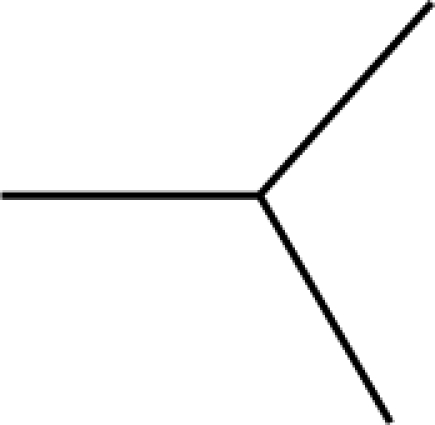
3	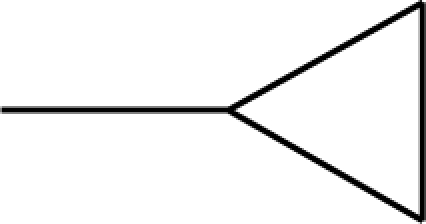	49	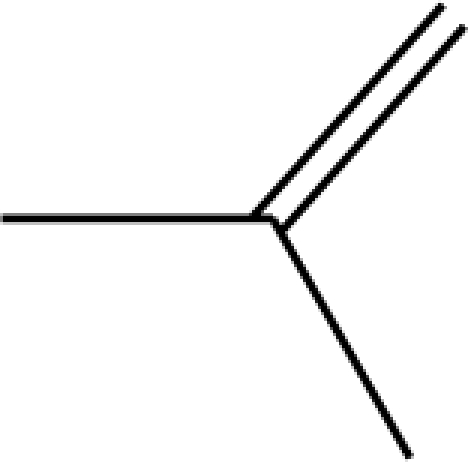
44	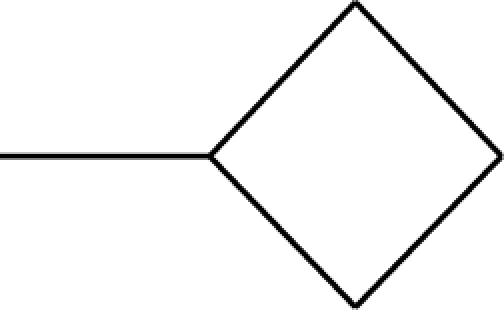	50*	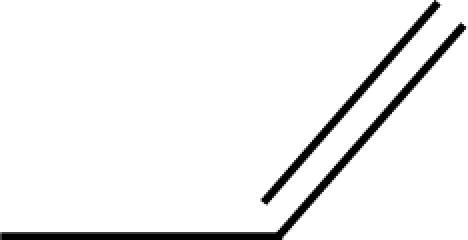
45*	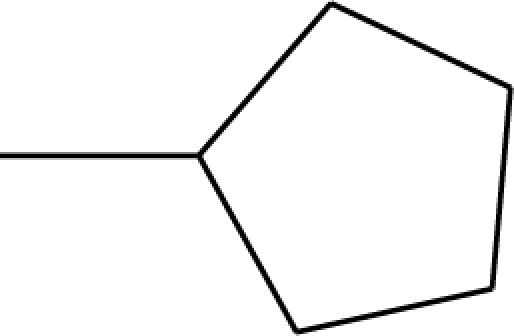	51	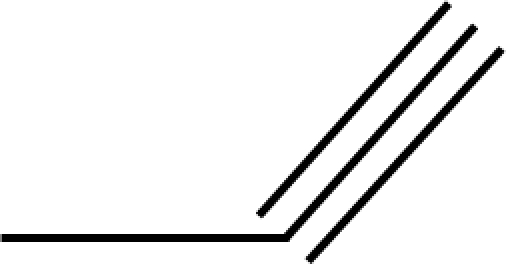
46	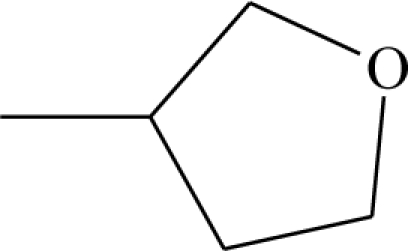	52	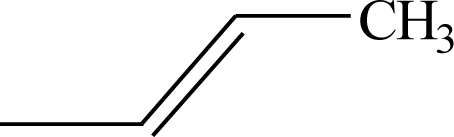
47	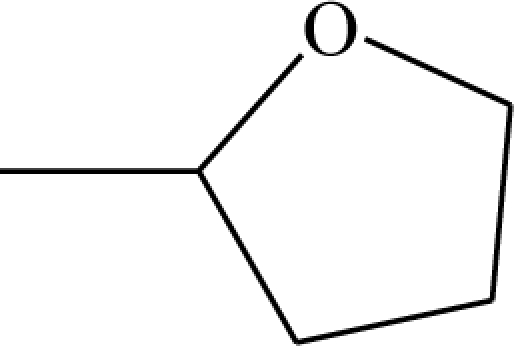	53*	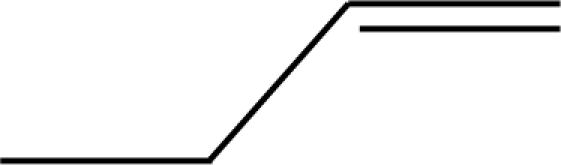

* -Test set.

**Table 2.** The atom numbers and three-atom sequences defining the two alignments (compound 43 is used to define the atom number).

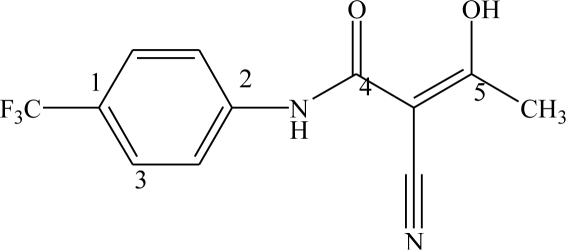			
**Alignment No.**	**1st atom**	**2nd atom**	**3rd atom**
1	1	2	3
2	2	4	5

**Table 3.** Statistics of the various SOMFA models.

	**Rat DHODH**						**Mouse DHODH**					

	All analogues		Aromatic substituted analogues		Side chain 3-substituted analogues		All analogues		Aromatic substituted analogues		Side chain 3-substituted analogues	

Align.	1	2	1	2	1	2	1	2	1	2	1	2
r^2^	0.658	**0.687**	**0.758**	0.665	0.778	**0.897**	0.517	**0.572**	0.554	**0.657**	0.697	**0.859**
r_CV_^2^	0.636	**0.664**	**0.739**	0.641	0.715	**0.865**	0.485	**0.545**	0.516	**0.620**	0.610	**0.822**
F	98.428	**112.251**	**125.687**	79.723	35.180	**87.269**	52.608	**65.608**	47.310	**72.892**	23.106	**61.314**
s	0.651	**0.623**	**0.528**	0.621	0.604	**0.412**	0.657	**0.619**	0.628	**0.551**	0.590	**0.402**
c_1_	0.695	**0.766**	**0.650**	0.769	0.800	**0.987**	0.531	**0.625**	0.429	**0.660**	0.934	**0.918**
r_pred_^2^	0.818	**0.717**	**0.571**	0.549	0.972	**0.981**	0.657	**0.679**	0.512	**0.693**	0.993	**0.991**

r^2^, Non cross-validated correlation coefficient; r_CV_^2^, Cross validated correlation coefficient; F, F-test value; s, standard error of estimate; c_1_, mixing coefficient of SOMFA model; r_pred_^2^, Predictive r^2^.

**Table 4.** Observed and predicted activities of 42 compounds in the training set.

Compd	**Rat DHODH**			**Mouse DHODH**		

	log(1/IC50)			log(1/IC50)		

	Observed	Predicted	Residual^a^	Observed	Predicted	Residual^a^
1	5.699	5.846	−0.146	5.801	5.888	−0.088
2	6.631	6.454	0.175	6.541	6.182	0.358
3	7.678	7.783	−0.103	7.328	7.385	−0.055
4	6.320	6.873	−0.553	6.280	6.545	−0.265
6	6.465	6.772	−0.312	5.523	6.447	−0.927
7	4.876	5.421	−0.541	4.780	5.501	−0.721
8	7.102	6.474	0.625	7.444	6.336	1.103
9	7.276	7.383	−0.103	7.377	7.114	0.265
11	8.301	8.067	0.232	7.000	6.723	0.277
12	7.796	6.736	1.063	6.380	5.957	0.422
13	8.523	8.018	0.501	7.051	6.885	0.164
14	7.886	7.509	0.380	6.352	6.350	−0.002
16	6.801	6.923	−0.124	4.821	6.026	−1.206
17	8.301	7.754	0.545	6.762	6.681	0.079
18	6.730	6.254	0.475	5.429	5.684	−0.254
19	5.100	6.006	−0.906	−	−	−
21	4.660	5.428	−0.768	4.500	5.275	−0.776
22	7.167	5.759	1.410	5.420	5.450	−0.030
23	4.851	5.292	−0.442	−	−	−
24	5.830	6.012	−0.182	5.429	5.703	−0.273
26	7.854	7.290	0.559	7.260	6.698	0.561
27	7.398	7.617	−0.217	7.149	6.984	0.165
28	7.959	8.423	−0.463	6.550	7.119	−0.567
29	7.4819	7.104	0.375	7.400	6.697	0.703
31	7.1029	7.043	0.056	6.550	6.543	0.007
32	7.3019	7.260	0.039	7.201	6.813	0.387
33	7.553	6.803	0.746	7.444	6.875	0.564
34	7.959	7.718	0.241	6.750	6.729	0.021
36	6.200	6.092	0.107	5.599	5.299	0.300
37	6.530	6.312	0.217	6.201	5.678	0.521
38	6.229	7.021	−0.791	6.250	6.438	−0.188
39	4.750	5.469	−0.720	5.301	5.380	−0.081
41	5.670	5.640	0.030	5.070	5.090	−0.020
42	5.830	6.735	−0.905	5.801	5.856	−0.055
43	7.886	7.906	−0.016	7.161	7.431	−0.270
44	6.550	6.402	0.147	6.680	6.274	0.406
46	4.750	5.791	−1.041	4.932	5.664	−0.732
47	4.350	5.299	−0.950	5.100	5.488	−0.388
48	6.530	6.261	0.268	7.036	6.210	0.826
49	6.600	6.371	0.229	6.710	6.276	0.434
51	6.301	6.505	−0.205	5.680	6.153	−0.473
52	5.851	6.039	−0.189	5.370	5.743	−0.373

a Residual = Observed − predicted.

**Table 5.** Observed and predicted activities of 11 compounds in the test set.

**Compd**	**Rat DHODH**			**Mouse DHODH**		

	log(1/IC50)			log(1/IC50)		

	Observed	Predicted	Residual^a^	Observed	Predicted	Residual^a^
5	7.201	6.882	0.318	7.444	6.569	0.871
10	5.343	6.928	−1.588	4.429	6.185	−1.755
15	6.080	7.117	−1.037	4.650	6.340	−1.690
20	7.678	7.376	0.304	7.301	6.998	0.302
25	6.801	6.593	0.207	5.951	6.084	−0.134
30	5.903	5.710	0.190	5.429	5.794	−0.364
35	7.745	8.034	−0.294	6.750	6.847	−0.097
40	6.750	6.632	0.118	6.201	5.895	0.305
45	4.500	5.313	−0.813	4.550	5.392	−0.842
50	7.638	6.461	1.179	6.750	6.086	0.664
53	6.971	6.298	0.672	7.201	6.174	1.026

a Residual = Observed − predicted.
